# Supplementary figures and images for: Transcriptional responses to chronic oxidative stress require cholinergic activation of G-protein-coupled receptor signaling
Source: eLife. 2026 Jun 8;14:RP107726. doi: 10.7554/eLife.107726 (PMC13246005; doi:10.7554/eLife.107726)

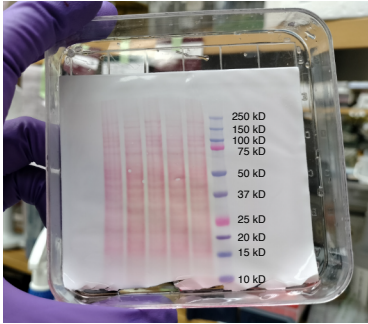

Ponceau S staining

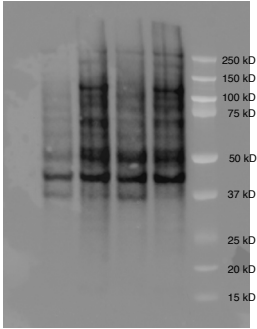

Chemiluminescence+colorimetry

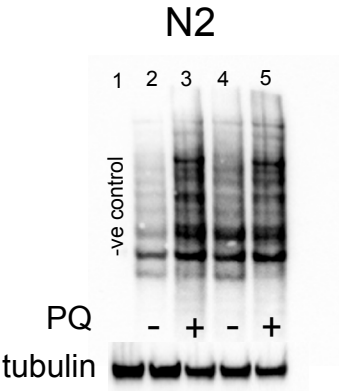

Chemiluminescence

Supplement: Figure 1—figure supplement 1—source data 2. [file elife-107726-fig1-figsupp1-data2.zip › figure 1 supplement 1 source data 3/labeledN2_PQ and control_48 hr.pdf]

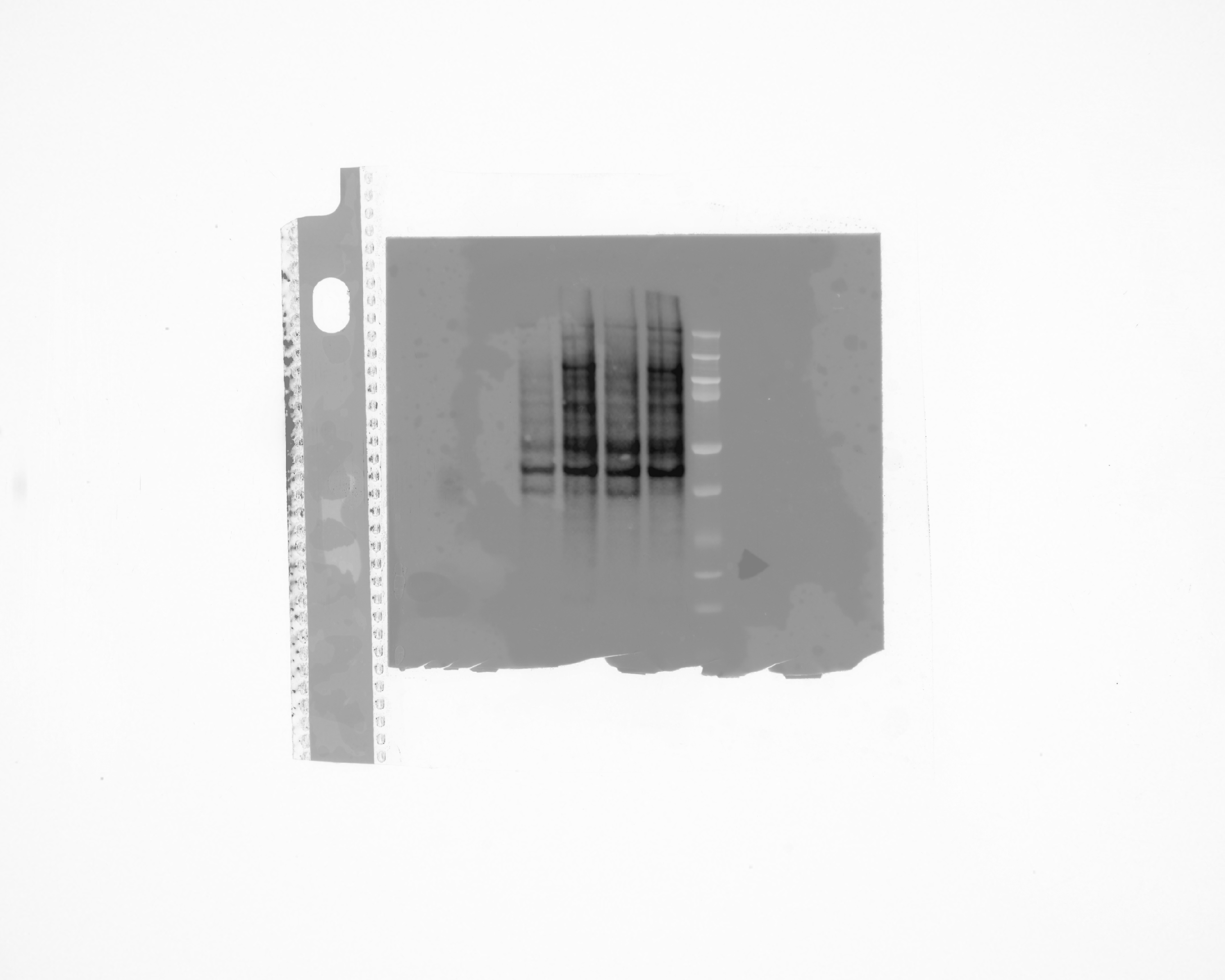

Supplement: Figure 1—figure supplement 1—source data 3. [file elife-107726-fig1-figsupp1-data3.zip › figure 1 supplement 1 source data 3/wtPQ4mM48h(Composite).tif]

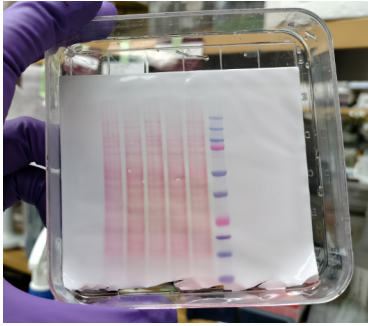

Ponceau S staining

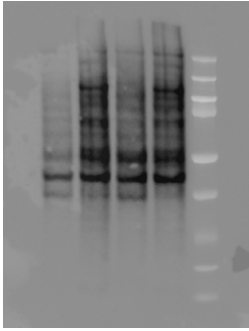

Chemiluminescence+colorimetry

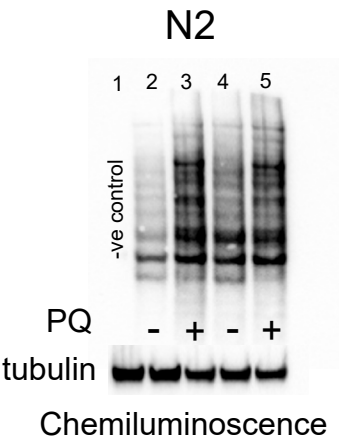

Supplement: Figure 1—figure supplement 1—source data 3. [file elife-107726-fig1-figsupp1-data3.zip › figure 1 supplement 1 source data 3/labeledN2_PQ and control_48 hr.pdf]

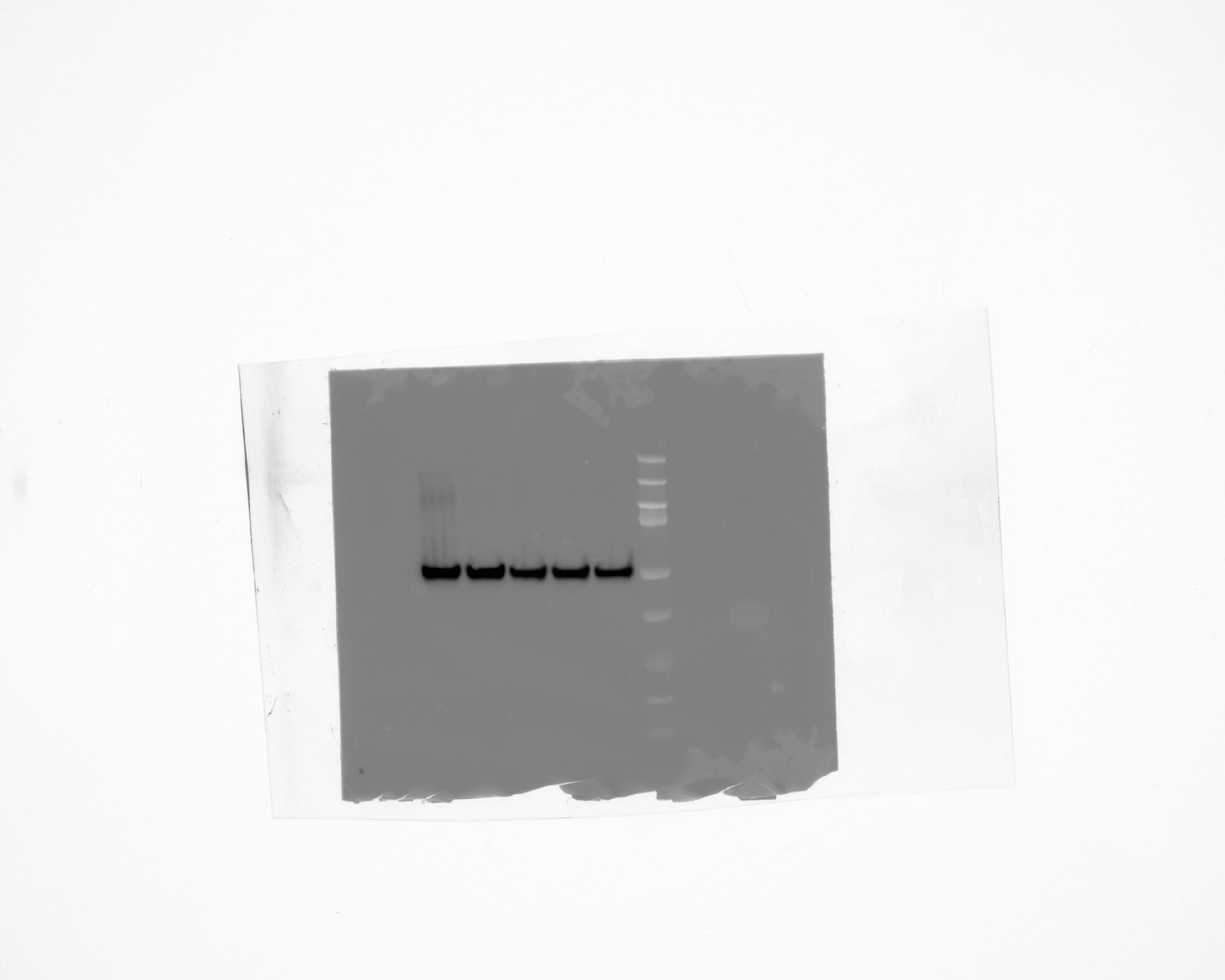

Supplement: Figure 1—figure supplement 1—source data 3. [file elife-107726-fig1-figsupp1-data3.zip › figure 1 supplement 1 source data 3/tubulin control for WT_PQ 48h(Composite).tif]
