# Supplementary material for: Transcriptional responses to chronic oxidative stress require cholinergic activation of G-protein-coupled receptor signaling
Source: eLife. 2026 Jun 8;14:RP107726. doi: 10.7554/eLife.107726 (PMC13246005; doi:10.7554/eLife.107726)
Supplement: Peer review history

# Editor report: eLife Assessment

**DOI:** 10.7554/eLife.107726.4.sa0

**Authors:** Paschalis Kratsios

**Affiliations:** University of Chicago United States

**Keywords:** Solid, Valuable

This **valuable** study advances our understanding of how organisms respond to chronic oxidative stress. Using the nematode *C. elegans*, the authors identified key neuronal signaling molecules and their receptors that are required for stress signaling and survival. The evidence supporting the conclusions is **solid**, including rigorous genetics, stress response analysis, and transcriptional profiling. This research will be of broad interest to neuroscientists and researchers working in the field of oxidative stress regulation.

# Referee report: Reviewer #2 (Public review)

**DOI:** 10.7554/eLife.107726.4.sa1

In this paper, Biswas et al. describe the role of acetylcholine (ACh) signaling in protection against chronic oxidative stress in *C. elegans*. They showed that disruption of ACh signaling in either unc-17 mutant or gar-3 mutants led to sensitivity to toxicity caused by chronic paraquat (PQ) treatment. Using RNA seq, they found that approximately 70% of the genes induced by chronic PQ exposure in wild type failed to upregulate in these mutants. The overexpression of gar-3 selectively in cholinergic neurons was sufficient to promote protection against chronic PQ exposure in an ACh-dependent manner. The study points to a previously undescribed role for ACh signaling in providing organism-wide protection from chronic oxidative stress likely through the transcriptional regulation of numerous oxidative stress-response genes. The paper is well-written, and the data are robust. While the study identifies the muscarinic ACh receptor gar-3 as an important regulator of the response to PQ, the specific neurons in which gar-3 functions were not unambiguously identified, and the sources of ACh that regulate GAR-3 signaling and the identities of the tissues targeted by gar-3 remain unknown.

Comments on revisions:

No further comments.

# Author comment: Author response

**DOI:** 10.7554/eLife.107726.4.sa2

**Authors:** Kasturi Biswas, Caroline Moore, Hannah Rogers, Khursheed A Wani, Arjamand Mushtaq, Read Pukkila-Worley, Daniel P Higgins, Amy K Walker, Gregory P Mullen, James B Rand, Michael M Francis

**Affiliations:** University of Massachusetts Chan Medical School worcester United States; University of Massachusetts Chan Medical School worcester United States; University of Massachusetts Chan Medical School worcester United States; University of Massachusetts Chan Medical School Worcester United States; University of Massachusetts Chan Medical School Worcester United States; University of Massachusetts Chan Medical School Worcester United States; University of Massachusetts Chan Medical School worcester United States; University of Massachusetts Chan Medical School Worcester United States; Oklahoma Medical Research Foundation Oklahoma City United States; Oklahoma Medical Research Foundation Oklahoma City United States; University of Massachusetts Chan Medical School Worcester United States

The following is the authors’ response to the previous reviews

**Public Reviews:**

**Reviewer #1 (Public review):**

Summary:

The researchers aimed to identify which neurotransmitter pathways are required for animals to withstand chronic oxidative stress. This work thus has important implications for disease processes that are caused/linked to oxidative stress. This work identified specific neurotransmitters and receptors that coordinate stress resilience, both prior to and during stress exposure. Further, the authors identified specific transcriptional programs coordinated by neurotransmission that may provide stress resistance.

Strengths:

The manuscript is very clearly written with a well-formulated rationale. Standard *C. elegans* genetic analysis and rescue experiments were performed to identify key regulators of the chronic oxidative stress response. These findings were enhanced by transcriptional profiling that identified differentially expressed genes that likely affect survival when animals are exposed to stress.

Weaknesses:

Where the gar-3 promoter drives expression was not discussed in the context of the rescue experiments in Fig 7.

Comments on revisions:

This issue has now been appropriately addressed in the revision.

We thank the reviewer for their time and constructive feedback.

**Reviewer #2 (Public review):**

In this paper, Biswas et al. describe the role of acetylcholine (ACh) signaling in protection against chronic oxidative stress in *C. elegans*. They showed that disruption of ACh signaling in either unc17 mutant or gar-3 mutants led to sensitivity to toxicity caused by chronic paraquat (PQ) treatment. Using RNA seq, they found that approximately 70% of the genes induced by chronic PQ exposure in wild type failed to upregulate in these mutants. The overexpression of gar-3 selectively in cholinergic neurons was sufficient to promote protection against chronic PQ exposure in an AChdependent manner. The study points to a previously undescribed role for ACh signaling in providing organism-wide protection from chronic oxidative stress likely through the transcriptional regulation of numerous oxidative stress-response genes. The paper is well-written, and the data are robust, though some conclusions seem preliminary and are not fully support the current data (see below). While the study identifies the muscarinic ACh receptor gar-3 as an important regulator of the response to PQ, the specific neurons in which gar-3 functions were not unambiguously identified, and the sources of ACh that regulate GAR-3 signaling and the identities of the tissues targeted by gar-3 were not addressed.

Comments on revisions:

The authors addressed my comments adequately in their revised submission. Please include representative images to accompany the quantification of the new results presented in Fig S4A.

We thank the reviewer for their time and constructive feedback. We now include representative images as requested.
